# Acute Spotted Fever Rickettsiosis among Febrile Patients, Cameroon

**DOI:** 10.3201/eid1003.020713

**Published:** 2004-03

**Authors:** Lucy M. Ndip, Donald H. Bouyer, Amelia P.A. Travassos Da Rosa, V.P.K. Titanji, Robert B. Tesh, David H. Walker

**Affiliations:** * University of Buea, Buea, South West Province, Cameroon; † University of Texas Medical Branch, Galveston, Texas, USA

**Keywords:** rickettsiae, chikungunya virus, flavivirus, alphavirus, *Rickettsia africae*, vector-borne diseases

## Abstract

Although potential arthropod vectors are abundant in Cameroon, acute febrile illnesses are rarely evaluated for arboviral or rickettsial infections. Serum samples from 234 acutely febrile patients at clinics in Tiko and Buea, Cameroon, were examined for antibodies to *Rickettsia africae* and African alphaviruses and flaviviruses. These serum samples did not contain antibodies against typhoid, and blood malarial parasites were not detected. Serum samples of 32% contained immunoglobulin M antibodies reactive with *R. africae* by immunofluorescence assay and were reactive with outer membrane proteins A and B of *R. africae* by immunoblotting. These findings established a diagnosis of acute rickettsiosis, most likely African tick-bite fever. Hemagglutination inhibition testing of the serum samples also detected antibodies to Chikungunya virus (47%) and flaviviruses (47%). High prevalence of antibodies to arboviruses may represent a major, previously unrecognized public health problem in an area where endemic malaria and typhoid fever have been the principal diagnostic considerations.

Cameroon is situated 2°–14° north of the equator; it has a vast tropical rain forest located 2°–5° north of the equator, which provides a good habitat for a variety of hematophagous arthropods. Vector-borne bacterial and viral diseases are rarely considered by local clinicians, whose primary diagnostic focus is on endemic malaria and typhoid fever. Chikungunya fever is an arboviral disease transmitted by mosquitoes of the genus *Aedes*; it occurs in neighboring Nigeria; and epidemics have been reported in Angola, Burundi, the Central African Republic, Kenya, Namibia, Senegal, South Africa, Tanzania, Uganda, and Zimbabwe (*1*–*3*). Antibodies to chikungunya virus (CHIKV) also were observed in German aid workers who had served in Bénin, Burkina Faso, and Zambia (*4*). The extent of CHIKV infection in the human population of Cameroon is unknown.

*Aedes aegypti* and *A.* *albopictus,* the major vectors of dengue fever, are both present in Cameroon (*5*,*6*). Although no information is available on the prevalence of dengue fever in Cameroon, epidemics of the disease have been reported in other neighboring African countries. Epidemic dengue hemorrhagic fever has not been reported in Africa, but sporadic cases clinically compatible with it have been reported in Mozambique and Djibouti (*7*). Yellow fever is endemic in much of sub-Saharan Africa, and large outbreaks of the disease have been reported in Ethiopia, Senegal, Nigeria, and Guinea (*8*,*9*). Although epidemics of yellow fever have not been reported in Cameroon, it is nonetheless considered to be a high-risk zone for the disease (*8*). Currently, no immunization programs are in place in the country to prevent yellow fever. Febrile illnesses such as chikungunya fever, dengue fever, and nonicteric yellow fever can be difficult to recognize, especially during the early stages of the disease and in a malaria-endemic zone (*2*).

*Rickettsia africae* is a spotted fever group rickettsia transmitted by *Amblyomma* ticks (*10*). It is endemic in some southern African countries, such as Zimbabwe and South Africa; most cases are reported in travelers returning from these countries (*11*,*12*). Although serologic surveys in Angola, Burkina Faso, Central African Republic, Congo, Ivory Coast, Mali, and Zimbabwe have previously detected antibodies to spotted fever group rickettsiae, the first human case of *R. africae* infection was not reported until 1992 (*13*–*15*). An earlier serologic survey that used a method less reliable than the immunofluorescent antibody assay demonstrated rickettsial antibodies in cattle and humans in the northern region of Cameroon and in other animals in the south of the country (*16*,*17*). Since then, little has been done to determine the incidence of rickettsioses in Cameroon. In this study, we sought to detect antibodies to spotted fever group rickettsiae, CHIKV, yellow fever, dengue, West Nile, and Spondweni viruses in serum samples collected from patients with symptoms of an acute febrile illness seen at clinics in the South West Province of Cameroon but in whom laboratory results for malaria and typhoid fever were negative.

## Materials and Methods

### Study Population

**Figure 1:**
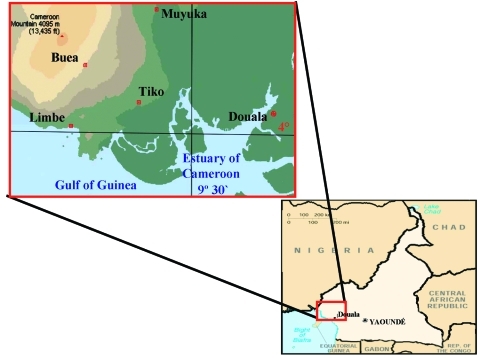
Location of townships in South West Province of Cameroon where samples were obtained.

A total of 234 serum samples were obtained at the Cameroon Development Corporation Central Clinic in Tiko (180 samples) and the Mount Mary Health Center in Buea (54 samples) from February 15 to March 31, 2001, from patients with clinical symptoms of a febrile illness and laboratory test results that excluded a diagnosis of malaria or typhoid fever. The samples were collected into sterile containers, and the serum samples were stored at –20°C. One hundred forty-two samples were from female patients, and 92 were from male patients; most samples were from adults (Table 1). The study participants were all residents of the South West Province of Cameroon from locations along the Atlantic Coast (Figure 1): Buea (4° 9′ N, 9° 13′ E), 52 patients; Limbe (4° 1′ N, 9° 12′ E), 27 patients; Muyuka (4° 10′ N, 9° 25′ E), 22 patients; and Tiko (4° 2′ N, 9° 19′ E), 133 patients. Yellow fever vaccine has not been administered routinely or in response to disease outbreaks in this region. The research protocol was approved by the Cameroon Ministry of Health and the administration of the clinics to ensure the ethical conduct of the study.

**Table 1:** Distribution of febrile Cameroonian patients in this study according to age and sex

Age group (y)	Female	Male	Total
<5	2	2	4
6–20	13	10	23
>21	127	80	207
Total	142	92	234

### Immunofluorescence Assay

Immunofluorescence assays (IFAs) were performed to detect antibodies to *R. africae* and *R. conorii*, as previously reported (*19*). Serial twofold dilutions (1 of 32 to 1 of 4,096) of human serum were prepared in phosphate-buffered saline (PBS) containing 1% bovine serum albumin (BSA) and 0.1% Tween 20. Antigen slides were blocked in PBS containing 1% BSA and 0.01% sodium azide. Ten microliters of each serum dilution was added to each well of the antigen slide and incubated for 30 min at 37°C in a humidity chamber. The slides were subsequently rinsed with a stream of PBS containing 0.1% Tween and then washed twice more in the same solution for 10 min. Fluorescein isothiocyanate–conjugated goat anti-human immunoglobulin (Ig) A, IgG, and IgM immune serum (KPL Inc., Gaithersburg, MD), 10 μL diluted 1 to 100 in PBS containing 1% BSA and 0.01% Tween 20, was added to each well and incubated in a humidity chamber for 30 min at 37°C. The slides were washed once in PBS containing 0.1% Tween 20 for 10 min and once with PBS containing 0.1% Tween 20 and 0.01% Evans blue for 10 min. The rinsed slides were blot dried, mounted with gel mount (Biomeda Corp, Foster City, CA) and observed under a fluorescence microscope at 400X magnification. Serum samples yielding distinctly fluorescent rickettsiae at a 1 to 64 dilution or higher were considered positive.

For assaying IgM antibody to *R. africae* and *R. conorii*, IgG was removed from IgG/IgA/IgM–positive serum by using recombinant protein G (MiniRapiSepM, PanBio, InDx, Baltimore, MD) according to the manufacturer’s instructions. Serum samples with specific fluorescence at >1:32 dilution were considered positive. Serial twofold dilutions of serum were tested to determine the endpoint titer.

### Western Immunoblot Assay

*R. africae* and *R. conorii* were released from Vero cell components by sonication and purified by density gradient centrifugation. Proteins were examined for immunoreactivity with the Cameroonian sera by Western immunoblotting (*20*,*21*).

### Preparation of Arbovirus Antigens

Arbovirus antigens were produced by sucrose-acetone extraction (*22*) of brains from newborn mice inoculated intracerebrally with the following arboviruses: CHIKV (Bili 4 strain), o’nyong-nyong virus (ONNV) (MP 30), yellow fever virus (17D), dengue 1 virus (Mochizuki strain), dengue 2 virus (New Guinea C), dengue 3 virus (H87), dengue 4 virus (H-241), West Nile virus (NY 385/99), and Spondweni virus (SAAR94). All arboviruses were obtained from the Arbovirus Reference Center at the University of Texas Medical Branch.

### Hemagglutination-Inhibition Test

Hemagglutination-inhibition (HI) tests were performed by using a microtechnique described previously (*22*). Before being tested, serum samples were treated with acetone to remove nonspecific inhibitors, and then goose erythrocytes were added to absorb natural agglutinins. CHIKV and ONNV antigens were tested at pH 6.2; dengue 1, 2, and 3 and yellow fever viruses were tested at pH 6.4; and dengue 4, West Nile, and Spondweni viruses were tested at pH 6.6. For the HI test, 4–8 U of antigen was used. Titers were recorded as the highest dilutions causing complete or almost complete inhibition of hemagglutination.

### Complement Fixation Test

Complement fixation (CF) tests were performed according to a microtechnique modified from Fulton and Dumbell (*23*), using 2 full U of guinea pig complement and the sucrose-acetone–extracted CHIKV and ONNV antigens described earlier. Serum samples were incubated at 60°C for 20 min before testing. Titers were recorded as the highest dilution giving 3+ or 4+ fixation of complement on a scale of 0 to 4+ (0 = complete hemolysis and 4+ = no hemolysis).

## Results

### Serologic Test for Rickettsial Antibodies

The 234 serum samples initially were screened by IFA for IgG, IgA, and IgM antibodies against *R. africae* and *R. conorii*; the samples containing antibodies at a titer of 32 were examined by the IgM-specific IFA. The results indicated that 75 patients (32.1%) had IgM antibodies reactive with *R. africae*. Fifty-one (35.9%) of the 142 female patients had IgM antibodies against *R. africae*; and 24 (26.1%) of the male patients had IgM anti–*R. africae* antibodies. The distribution of endpoint IgM titers for *R. africae* was 32 (24%), 64 (18%), 128 (20%), 256 (14.4%), 512 (12%), 1,024 (8%), and 2,048 (2.7%).

**Figure 2:**
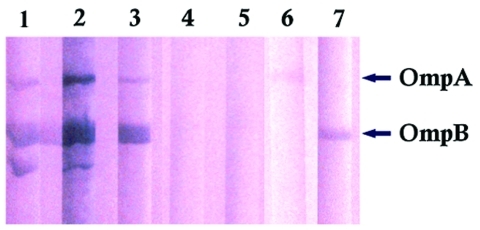
Immunoblot of *Rickettsia africae* antigens with *R. africae–*positive and –negative patient serum samples. Lanes 1–3: *R. africae–*positive patients’ serum samples; lanes 4–5: *R. africae–*negative patients’ serum samples; lane 6: anti–OmpA monoclonal antibody; lane 7: anti–OmpB monoclonal antibody.

The distribution of IgM titers against *R. conorii* among the 75 *R. africae*–positive serum samples was 32 (29.3%), 64 (32.8%), 128 (17.2%), 256 (19%), and 512 (1.7%). Twenty-six of the samples had antibody titer fourfold or greater for *R. africae* than for *R. conorii*. None of the serum samples had titers fourfold higher for *R. conorii.* Nineteen serum samples had antibody titers twofold higher for *R. africae*; three had antibody titers twofold higher for *R. conorii.* Eight serum samples had a titer of 32 for *R. africae* and were negative for *R. conorii.* Nineteen samples had the same titers for *R*. *africae* and *R. conorii*. Of the patients with IgM antibodies to spotted fever group rickettsiae, 67 (89%) were adults, and 8 (11%) were <20 years of age. None of four children <5 years of age had IgM antibodies to *R. africae.* Western immunoblot analysis confirmed the IFA results (Figure 2). The reactive serum samples contained antibodies that were immunoreactive with both OmpA and OmpB of *R. africae*.

### Serologic Testing for Arboviral Antibodies

HI testing of serum samples from febrile patients in South West Province demonstrated antibodies to CHIKV, dengue 1–4, yellow fever, West Nile, and Spondweni viral antigens (Table 2). Antibodies were detected in some of the serum samples for all of the viruses tested, and considerable cross-reactivity among the flaviviruses was observed. The HI tests with dengue 1–4 viruses and yellow fever virus antigens yielded similar results; titers ranged from 20 to 2,560. HI antibodies to CHIKV antigen were detected in 103 (44%) of the 234 serum samples, and a titer of ≥1,280 was observed in 11 samples. Comparative CF testing demonstrated that the endpoint titers were higher against CHIKV antigen than ONNV antigen, a finding that suggests that CHIKV was the infecting alphavirus.

**Table 2:** Geographic origin of 234 Cameroonian patients with antibodies to chikungunya, yellow fever, dengue, West Nile, and Spondweni viruses^a^

Antigen	Buea (n = 52)	Limbe (n =27)	Muyuka (n = 22)	Tiko (n = 133)	Total	Geometric mean titer^b^	
Chikungunya	15	18	5	65	103	250	
Yellow fever	12	10	4	67	93	56	
Dengue 1	5	11	5	59	80	91	
Dengue 2	6	9	5	55	75	66	
Dengue 3	6	6	3	41	56	71	
Dengue 4	4	6	3	42	55	85	
West Nile	0	9	2	51	62	71	
Spondweni	0	9	3	53	65	77	

^a^As detected by hemagglutination inhibition test. ^b^Reciprocal of titers.

Eighty-four (35.9%) of the 234 serum samples contained HI antibodies reactive with one or more of the dengue 1–4 viruses, 93 (39.7%) had antibodies to yellow fever virus, 62 (26.5%) contained antibodies against West Nile virus, and 65 (27.8%) had antibodies to Spondweni virus. In all, 110 (47%) of the serum samples contained antibodies to one or more of the flaviviruses. Antibodies to CHIKV and the flaviviruses were detected in patients from each of the study locations (Table 2).

## Discussion

In Cameroon, as in many other African countries, rickettsioses and arboviral infections are rarely considered when evaluating patients with acute, undifferentiated febrile illnesses. This situation can be attributed in part to unavailability of specific laboratory tests, equipment, and expertise and also the limited economic resources in many countries of the region. In Cameroon, most patients are evaluated by clinical laboratory methods only for malaria and typhoid fever. Since many patients with rickettsial and arboviral illnesses initially have acute febrile syndromes, their diagnosis is difficult without confirmatory laboratory tests.

Previous retrospective serologic surveys detected antibodies to rickettsiae in Zimbabwe, Ivory Coast, Central African Republic, and Egypt (*15*,*24*–*27*). However, detecting IgM antibodies to rickettsiae by IFA, in association with febrile illness, is a better approach to identifying recent or current infections; Western immunoblot assay can be used to confirm positive IFA results.

Our results indicate that one or more rickettsioses are endemic in Cameroon. Most previous serologic surveys have detected antibodies in healthy study participants (*14*,*17*–*19*,*27*); however, our study population was composed of patients with acute febrile illnesses other than malaria or typhoid fever. The specific cause of the agent that stimulated the antibodies to spotted fever group rickettsiae in these patients remains to be determined. Raoult et al. (*11*) reported spotted fever rickettsiosis in travelers returning to Europe from Africa with documentation of *R. africae* infections by isolation of the agent and specific polymerase chain reaction results. The same authors also evaluated serologic methods to distinguish between infection with *R. africae* and *R. conorii*; they reported that a fourfold or greater titer to *R. africae* by IFA had a specificity of 100% but a sensitivity of only 26% (*11*). This criterion supported the diagnosis of *R. africae* infection in 26 (35%) of the 75 patients in our study with acute rickettsiosis and did not establish a diagnosis of *R. conorii* infection in any patient. Our immunoblot detection of antibodies to *R. africae* OmpA and OmpB also supported the diagnosis of *R. africae* infection rather than *R. conorii.* However, cross-reactivity of the antibodies with OmpA of another rickettsial organism that was not included in the study, including any undiscovered rickettsiae, is possible. Raoult et al. (*11*) have diagnosed *R. africae* infections in individual travelers whose infections were acquired in nearby Central African Republic and Gabon. Although our findings suggest that the infections in Cameroonian patients were caused by *R. africae*, the possibility of the occurrence of another spotted fever group rickettsial infection cannot be excluded because spotted fever group rickettsiae share closely related antigens. Moreover, the most reliable and specific method to establish the identity of the causative agent is isolating it from the blood or tissue of suspected patients; this remains to be achieved in Cameroon. Other problems that need to be addressed include a full description of the clinical spectrum of this rickettsiosis in African patients, the risk factors for severe illness, the vector(s), natural history of the bacterium, and epidemiology of the disease.

After an incubation period averaging 6–7 days, *R. africae*–infected travelers returning to Europe and North America manifested an influenza-like syndrome including fever (88%), myalgia (63%), eschars (95%), regional lymphadenopathy (43%), and rash (46%) in one series (*11*). A subsequent series of Norwegian travelers to sub-equatorial Africa had a similar syndrome of fever, headache, and myalgia but with a lower proportion of patients with an eschar (53%) (*28*). The low proportion of children in our study suggests that severity of the clinical manifestations may be age-dependent, as has been documented for Rocky Mountain spotted fever and louse-borne typhus fever (*29*). To date, African tick-bite fever has been characterized as a mild illness. However, the presence of glucose-6-phosphate dehydrogenase deficiency and the empiric treatment of the febrile illness with sulfonamide antimicrobials, both frequent situations in Africa, might result in more severe disease; this possibility needs to be evaluated (*30*–*33*). It could be predicted that *A. variegatum* might transmit *R. africae*, and that *Rhipicephalus* species would transmit *R. conorii*; but the susceptibility of other ticks in Cameroon to these rickettsiae has not been studied. The unexpectedly high incidence of spotted fever rickettsiosis in this population also suggests that the course of illness in many febrile patients in Cameroon might be ameliorated by early treatment with an antirickettsial drug such as doxycycline.

CHIKV infection is common in sub-Saharan Africa; antibodies to CHIKV have frequently been detected during serosurveys throughout the humid forest and semi-arid savannas of Africa (*1*,*2*,*34*–*36*). Although CHIKV and ONNV are closely related (*34*), our serum samples containing antibodies to alphaviruses yielded much lower titers against the ONNV antigen, suggesting that cross-reactivity was at a low level and that CHIKV was the circulating agent. Chikungunya fever is characterized by fever, headache, nausea, vomiting, myalgia, rash, and arthralgia (*35*). These clinical symptoms are similar to those of dengue viral infection and can lead to misdiagnosis (*37*). Evidence suggests that CHIKV circulates continually in sylvatic cycles in Africa; the virus has been isolated from forest-dwelling mosquitoes in several African countries including Senegal, Ivory Coast, and South Africa (*35*,*36*).

Dengue fever is endemic in tropical and subtropical regions worldwide. The possibility that Cameroon is another dengue-endemic region would not be surprising. This infection, which usually manifests as undifferentiated fever, can lead to hospitalization of large numbers of people. Outbreaks cause illness and death rates with substantial socioeconomic impact. The results of our study indicate that rickettsial and arboviral infections are common among residents of Cameroon and that local health personnel should include them in their differential diagnosis. For both the arboviral and rickettsial agents, much work remains to be done, particularly identification of the viruses and rickettsiae in patients and arthropods.
